# Regional Brain Responses Are Biased Toward Infant Facial Expressions Compared to Adult Facial Expressions in Nulliparous Women

**DOI:** 10.1371/journal.pone.0166860

**Published:** 2016-12-15

**Authors:** Bingbing Li, Gang Cheng, Dajun Zhang, Dongtao Wei, Lei Qiao, Xiangpeng Wang, Xianwei Che

**Affiliations:** 1 School of Psychology, Southwest University, Chongqing, China; 2 Center for Mental Health Education, Southwest University, Chongqing, China; 3 School of Educational Science, Guizhou Normal University, Guiyang, China; 4 Key Laboratory of Cognition and Personality (SWU), Ministry of Education, Chongqing, China; 5 Monash Alfred Psychiatry Research Centre, The Alfred and Central Clinical School, Monash University, Melbourne, Australia; Universita degli Studi di Udine, ITALY

## Abstract

Recent neuroimaging studies suggest that neutral infant faces compared to neutral adult faces elicit greater activity in brain areas associated with face processing, attention, empathic response, reward, and movement. However, whether infant facial expressions evoke larger brain responses than adult facial expressions remains unclear. Here, we performed event-related functional magnetic resonance imaging in nulliparous women while they were presented with images of matched unfamiliar infant and adult facial expressions (happy, neutral, and uncomfortable/sad) in a pseudo-randomized order. We found that the bilateral fusiform and right lingual gyrus were overall more activated during the presentation of infant facial expressions compared to adult facial expressions. Uncomfortable infant faces compared to sad adult faces evoked greater activation in the bilateral fusiform gyrus, precentral gyrus, postcentral gyrus, posterior cingulate cortex-thalamus, and precuneus. Neutral infant faces activated larger brain responses in the left fusiform gyrus compared to neutral adult faces. Happy infant faces compared to happy adult faces elicited larger responses in areas of the brain associated with emotion and reward processing using a more liberal threshold of p < 0.005 uncorrected. Furthermore, the level of the test subjects’ Interest-In-Infants was positively associated with the intensity of right fusiform gyrus response to infant faces and uncomfortable infant faces compared to sad adult faces. In addition, the Perspective Taking subscale score on the Interpersonal Reactivity Index-Chinese was significantly correlated with precuneus activity during uncomfortable infant faces compared to sad adult faces. Our findings suggest that regional brain areas may bias cognitive and emotional responses to infant facial expressions compared to adult facial expressions among nulliparous women, and this bias may be modulated by individual differences in Interest-In-Infants and perspective taking ability.

## Introduction

Baby schema serves as an “innate releasing mechanism”, a fundamental social instinct that serves to initiate and maintain a parent-infant relationship [1]. A number of imaging studies have investigated the biological basis for this mechanism by comparing brain responses to neutral infant facial expressions to those of neutral adult facial expressions. Compared to unfamiliar adult faces, unfamiliar infant faces activate the fusiform gyrus (FFG) and precuneus (PCU), which are involved in face processing, attention, theory of mind (ToM), and empathy [2]. Kringelbach [3] reported that unfamiliar infant faces compared to carefully matched unfamiliar adult faces elicited greater activation in the medial orbitofrontal cortex (OFC), which is associated with reward processing. Another recent functional magnetic resonance imaging (fMRI) study revealed that infant faces evoked stronger activation in motor-related areas than matched adult faces, including the lateral premotor cortex and supplementary motor area, and no difference in brain activation is observed compared adult faces to infant faces [4]. Activation of motor-related regions in response to infant faces suggests preparation by adults for communicative behavior with infants as well as attachment and caregiving. These studies thus have uncovered the biological basis for the ‘‘innate releasing mechanism” for affection and nurturing of young infants, as described by Lorenz [5].

Infant facial expressions convey salient information about their emotional states and needs. Accurate interpretation and sensitive caregiving response to the needs underlying infant facial expressions may be critical to infant development [6]. Pechtel [7] reviewed recent fMRI findings and elaborated on the neural components associated with internal maternal processes to infant cues. For example, Lorberbaum [8] found that infant crying activates the cingulate cortex and thalamus (THA) in women, which is consistent with MacLean’s thalamocingulate theory of maternal behavior [9]. Another fMRI study demonstrated that happy infant faces compared to neutral infant faces activated reward-related brain areas, such as the ventral striatum, caudate, and ventromedial prefrontal and orbitofrontal cortices. Furthermore, sad infant faces compared to neutral infant faces activated the PCU, cuneus, and posterior cingulate cortex (PCC), which are implicated in empathic processing [10].

It is interesting to note that neural responses in mothers are specific to the processing of infant facial expressions rather than a more generalized bias to any emotional expressions. Specifically, in an event-related potential (ERP) study, early latency of the N1 response was observed for strong intensity facial expressions compared to mild intensity facial expressions, which reflected faster emotional recognition performance. In addition, the early posterior negativity (EPN) waveform is further enhanced by negative infant facial expressions. Neither of these effects is significant in ERPs elicited by adult facial expressions [11]. Using near-infrared spectroscopy, Nishitani found that infant facial emotion discrimination increased relative oxyhemoglobin concentrations in the right prefrontal cortex of mothers compared with non-mothers, whereas no differences were observed while viewing adult facial expressions [12]. The pattern of these findings indicates that motherhood biases brain responses to infant facial expressions, which raises the question as to whether infant facial expressions evoke larger brain responses than adult facial expressions.

The objective of the present study was to explore whether brain response is biased toward unfamiliar infant faces compared to unfamiliar adult faces in nulliparous women, which could further inform our understanding of the neural mechanisms underlying women’s internal processing of caregiving behavior. Previous fMRI studies have shown that happy adult facial expressions activate the basal ganglia, including the ventral striatum and putamen [13–15], whereas sad adult facial expressions activate the left amygdala and right temporal pole [16]. In the present study, we performed event-related fMRI in a relatively large sample of nulliparous women who were presented with a stream of unfamiliar infant and adult facial expression stimuli. We hypothesized that uncomfortable infant faces compared to sad adult faces would evoke larger responses in the FFG, PCU, PCC, and THA, and that happy infant faces would elicit greater activity compared to happy adult faces in reward-related regions.

We also examined whether individual differences in Interest-In-Infants and empathy were associated with different patterns of brain response to infant versus adult facial expressions. Freeman [17] recently proposed a neural network for flexible social perception, in which perceptual representations of faces in the FFG may be modulated by higher order social cognitive processes (such as stereotypes, attitudes, and goals). For example, persons who held a negative stereotypical opinion of dark-skinned people had a greater bias to perceive black faces as “Angry” [18]. Females tend to be more involved in infant care and are generally more interested in infants than are males [19, 20]. Therefore, we predicted that the FFG response to infant facial expressions would reflect individual differences in Interest-In-Infants. The PCU has been implicated in empathic processes [21] as well as attribution of emotion to infant facial expression by nulliparous women [10]. We further investigated the relationship between the PCU response bias to uncomfortable infant facial expressions and empathy in nulliparous women by correlation and regression analyses in the present study.

## Materials and Methods

### Participants

Thirty-five native Chinese-speaking right-handed nulliparous women gave informed written consent and participated in this study, which was approved by the Ethics Committee of Southwest University (NO. 2014179). All research was conducted in accordance with the Declaration of Helsinki. Three subjects exhibited excessive movement during multiple fMRI runs and were excluded from analyses. The remaining 32 subjects were between the ages of 18 and 25 years old (21.9 ± 1.8 years, mean ± standard deviation), in good health with no history of psychiatric or neurological disorders, and had normal or corrected-to-normal vision.

### Questionnaires and Stimuli

#### Interpersonal reactivity index

All subjects completed the Interpersonal Reactivity Index-Chinese (IRI-C) [22], which was used to evaluate empathic ability. This questionnaire includes 22 self-report items that measure four domains related to empathy and the representation of others’ mental states: Empathic Concern, Perspective Taking, Fantasy, and Personal Distress. Each item is rated on a 5-point Likert scale ranging from 0 (does not describe me well) to 4 (describes me very well). A higher score on each subscale indicates higher functioning for each aspect of empathy. Cronbach’s alpha coefficient for the total IRI-C and the four subscales ranged from 0.532 to 0.758, and the test-retest reliability ranged from 0.625 to 0.737. The accumulation of correlated variance on the IRI-C was 46.342% and had significant differentiation validity (P < 0.001) [22]. Thus, the Chinese adaptation of the IRI has good reliability and validity.

#### Interest in infants

Interest-In-Infants [23] was assessed with a Chinese version of the questionnaire [24]. On the Interest-In-Infants questionnaire, subjects are presented with the following scenario “If you were at a party and there was a baby in the room that you did not know, what would you most likely do?” Ten different types of interactions are presented (e.g., “ask to hold the baby”, “avoid the baby entirely”). Participants responded based on the likelihood of their engaging in each of the listed activities on a 6-point scale from 1 (not at all likely) to 6 (very likely). Items indicating avoidance of the infant were reverse-coded. Scores were obtained by summing the scores for each item, with higher scores reflecting more Interest-In-Infants.

#### Stimuli

Photographs of infant faces (ages 3–6 months old) were adapted from the Chinese Infant Affective Face Picture System, which was established by our group [25]. All infant photographs were obtained from a professional photography studio that specializes in infant photography. All photographs were included with permission of the parents. Adult faces (ages 17–23 years) were adapted from the Chinese Affective Face Picture System [26]. Adult photographs were collected from students in the acting and directing departments of two art colleges. All images were normalized to the same luminance and contrast using the SHINE toolbox in MATLAB (The MathWorks, Inc., Natick, MA, USA) [27]. Based on the findings of a previous study [28], we matched the infant facial expression of uncomfortableness with the adult facial expression of sadness. Infant and adult emotional expression intensity was rated from 1 (very weak) to 9 (very strong) by two independent groups of female college students (15 individuals in each group). Neutral faces were rated for the level of ‘neutrality’ the same as emotional faces were rated for emotional intensity. We were careful to select happy, neutral, and uncomfortable/sad faces of 15 infants and 15 adults that were matched for intensity. Fifteen face images were collected for each of the six conditions of interest (happy infant, neutral infant, uncomfortable infant, happy adult, neutral adult, and sad adult), resulting in a total of ninety images. All images were presented on a black background.

### Experimental Design for fMRI

**Fig 1:**
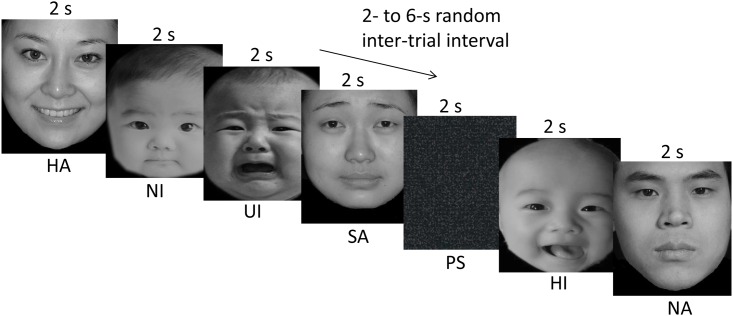
Examples of face images and probe stimuli presented during the fMRI experiment. Six types of face images are presented to subjects during the experiment: happy infant (HI), neutral infant (NI), uncomfortable infant (UI), happy adult (HA), neutral adult (NA), and sad adult (SA). A blank gray image is presented 5 times during each session as a probe stimulus. Randomized images are presented for 2 seconds with a random inter-stimulus interval of 2, 4, or 6 seconds.

Subjects viewed 60 intensity-matched face images (ten images for each condition of interest) on each of four functional runs. Images were pseudo-randomized within and between each run but not between subjects. A probe stimulus (a gray image), which subjects were instructed to respond to with a button press, was presented 5 times during each run to maintain the subject’s attention during the task; data from these stimuli were not included in data analyses. All images (face images and probe stimuli) were displayed foveally at the fixation point for 2 seconds with a random inter-stimulus interval of 2, 4, or 6 seconds (Fig 1). Before the experiments started, participants completed training sessions to familiarize themselves to the task. All participants gave written informed consent to publish these case details.

### fMRI Acquisition and Analysis

Functional images were acquired from a 3T Siemens Magnetom Trio Tim B17 MRI scanner (Siemens Medical Systems, Erlangen, Germany). T2*-weighted gradient echo planar imaging sequences were used to obtain functional images. A total of 788 volumes were collected from each subject. Each volume included 33 axial and interleaved acquired slices with 3-mm thickness and 0.6-mm gap oriented parallel to the anterior commissure-posterior commissure (AC-PC) plane (repetition time = 2000 ms; echo time = 30 ms; field of view = 200 × 200; 64 × 64 matrix; and flip angle = 90°). High-resolution T1-weighted images, which were composed of 176 volumes, were acquired for each subject for anatomical reference (repetition time = 1900 ms; echo time = 2.52 ms; slice thickness = 1 mm; field of view = 256 × 256; and voxel size = 1 × 1 × 1 mm^3^).

Data analyses were performed with Statistical Parametric Mapping version 8 (SPM 8; http://www.fil.ion.ucl.ac.uk/spm/software/spm8/) in MATLAB version 7.6 (The MathWorks Inc., Natick, MA, USA). Functional images were realigned for head movement using the median image as a reference, co-registered to the high-resolution structural image, and normalized to the T1 template in the Montreal Neurological Institute (MNI) space. Functional images were resampled to a voxel size of 3 x 3 x 3 mm^3^ and smoothed with a 4-mm full width at half maximum (FWHM) Gaussian kernel.

A two-stage random-effects model was conducted for random-effects analysis. In the first-level analysis, for each participant and session, six conditions (happy infant, neutral infant, uncomfortable infant, happy adult, neutral adult, and sad adult) from our experiment were modeled as separate regressors using a general linear model. Six head-motion parameters were modeled as independent covariates. All regressors were convolved by a canonical hemodynamic response function, and functional data were high-pass filtered to eliminate low-frequency noise. Six t-contrasts that were created (1 for one condition, and 0 otherwise) to generate six statistical parametric maps for each condition. These parametric maps were subsequently submitted to a group-level random-effects analysis.

Group-level analyses tested whether emotion type modulates neural activations associated with face type. We entered all six statistical parametric maps of each condition from each subject into a full two-factorial repeated-measures ANOVA that included two within-subjects factors (face type and emotion type). Results for main effects were thresholded at a peak-level family-wise error (FWE) corrected p < 0.05 (voxel-wise uncorrected p < 0.001). Results for the interaction effect were thresholded at a peak-level false discovery rate (FDR) corrected p < 0.05 (voxel-wise uncorrected p < 0.001) with an extent threshold of k = 10.

Simple effects were examined using t-contrasts with thresholds at a cluster-level FWE corrected p < 0.05 (voxel-wise uncorrected p < 0.001) for the uncomfortable and neutral face comparisons, but a more liberal threshold voxel-wise uncorrected p < 0.005 was applied to the comparison of happy infant faces to happy adult faces.

### Questionnaire Scores and Correlations with Brain Activity

For clusters exhibiting significant results, we created regions of interest (ROI; 6-mm radius sphere) centered on each peak coordinate (left FFG -21, -72, -6; right FFG 33, -62, -10; PCU 0, -57, 60), and extracted individual mean beta values of each ROI using MarsBar software [29]. Scores greater than 3 s.d. from their group mean were considered outliers and removed from the behavioral and fMRI data sets. This resulted in the removal of one beta value of the PCU and one empathic concern score. Therefore, correlation analysis included data from 30 participants.

To test our hypotheses, Pearson correlations were calculated between the bilateral FFG beta values and the Interest-In-Infants test score, and between the PCU beta values and IRI-C Likert scores. Then, a multiple regression analysis was conducted to determine which IRI-C factors predict the beta values of the PCU. All predictor variables were added simultaneously using the enter method.

## Results

### Validation of Infant and Adult Facial Expression Stimuli

The matched intensity ratings were as follows: sad/uncomfortable (5.99 ± 0.48 versus 6.22 ± 0.95, adult versus infant, respectively; p = 0.903); neutral (5.88 ± 0.03 versus 5.88 ± 0.05, adult versus infant, respectively; p = 0.983); and happy (6.55 ± 0.23 versus 6.52 ± 0.10, adult versus infant, respectively; p = 0.412). A between-subjects analysis of variance (ANOVA) of the intensity ratings demonstrated that the main effect of emotion (happy vs sad) was significant [F (1, 56) = 5.34, p < 0.05], but that the main effect of face type [F (1, 56) = 0.29, p = 0.59] and the interaction between emotion type and face type [F (1, 56) = 0.50, p = 0.48] did not reach significance.

The arousal of faces was rated by an independent group on a scale of 1 (the lowest) to 9 (the highest). A between-subjects ANOVA of the arousal ratings demonstrated that the main effects of emotion types [F (2, 84) = 164.0, P < 0.01], face types [F (1, 84) = 34.2, P < 0.01], and the interaction effect [F (2, 84) = 9.0, P < 0.01] all reached statistical significance. The multiple comparison of emotion types revealed significant arousal difference between happy and neutral facial expressions (M = 6.3, SE = 0.44 vs M = 3.8, SE = 0.76, P < 0.01), happy and sad facial expressions (M = 6.3, SE = 0.44, vs M = 4.9, SE = 0.73, P < 0.01) and sad and neutral facial expressions (M = 4.9, SE = 0.73, vs M = 3.8, SE = 0.76, P < 0.01). The simple effect test of interaction showed significant arousal differences between uncomfortable infant and sad adult expressions (M = 5.5, SE = 0.41 vs M = 4.4, SE = 0.60, P < 0.01) and neutral infant and adult facial expressions (M = 4.3, SE = 0.76 vs M = 3.3, SE = 0.41, P < 0.01), but not between happy infant and adult facial expressions (M = 6.3, SE = 0.31 vs M = 6.3, SE = 0.56, P = 0.95).

### Main Effects of Face and Emotion Type

**Fig 2:**
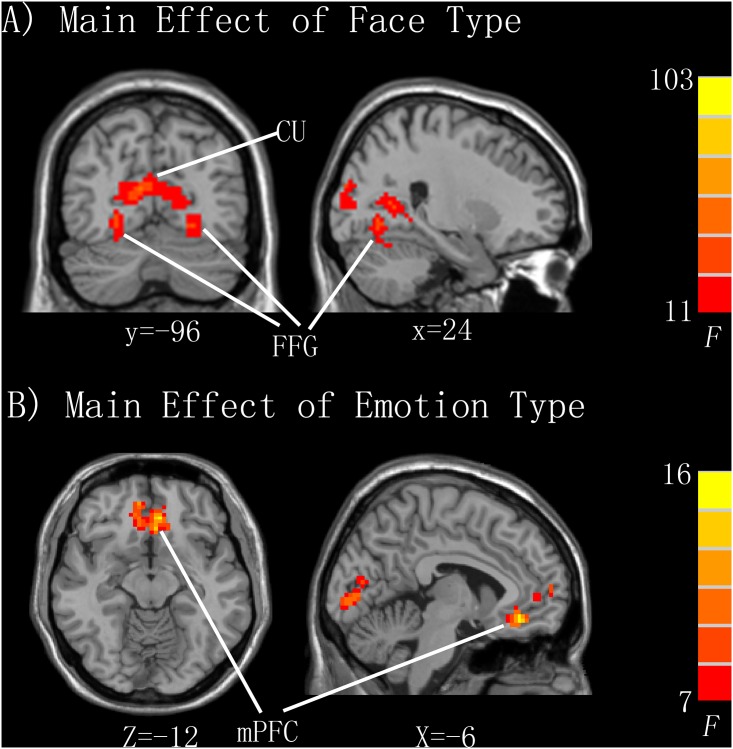
Activation regions of face and emotion processing. A) Main effect of face types. Fusiform gyrus (FFG) activity is greater in the processing of infant faces than adult faces, whereas cuneus (CU) activity is greater in the processing of adult faces than infant faces. For illustration, a voxel-wise p < 0.001 (uncorrected) and an extent threshold of k = 30 is used. B) Main effect of emotion type (happy, neutral, and sad). The medial prefrontal cortex (mPFC) is activated in the processing of emotion, with a cluster extending to the medial orbitofrontal cortex (OFC) and anterior cingulate cortex (ACC). For illustration, a voxel-wise p < 0.001 (uncorrected) and an extent threshold k = 100 is used.

Group analysis revealed a significant main effect of face type. Activation of the bilateral cuneus was higher for adult faces than infant faces, whereas activation of the bilateral fusiform and right lingual gyrus was higher for infant faces than adult faces (Table 1; Fig 2A). Group analysis also revealed a significant main effect of emotion type in the medial prefrontal gyrus, medial OFC, anterior cingulate cortex (ACC), and middle occipital gyrus (Table 1; Fig 2B).

**Table 1:** Brain activity associated with the main effects of face and emotion types.

Area	k	F Score	x	Y	Z
**Main Effect of Face Type**					
Adult face > Infant face					
R Occipital lobe/Cuneus	1142	103.2	9	-96	15
Infant face > Adult face					
R FFG	38	33.9	24	-69	-6
	31	29.7	36	-45	-9
L Occipital lobe/FFG	64	39.8	-21	-72	-6
R Lingual gyrus	11	30.1	6	-75	-3
**Main Effect of Emotion Type**					
R mPFC/mOFC/ACC	247	16.5	-6	27	-12
Middle occipital gyrus	132	16.5	-24	-93	3

Data are thresholded at a peak-level FWE-corrected p < 0.05 (voxel-wise uncorrected p < 0.001). R, right; L, left; FFG, fusiform gyrus; mPFC, medial prefrontal cortex; mOFC, medial orbitofrontal cortex; ACC, anterior cingulate cortex.

### Interaction between Face and Emotion Type

**Fig 3:**
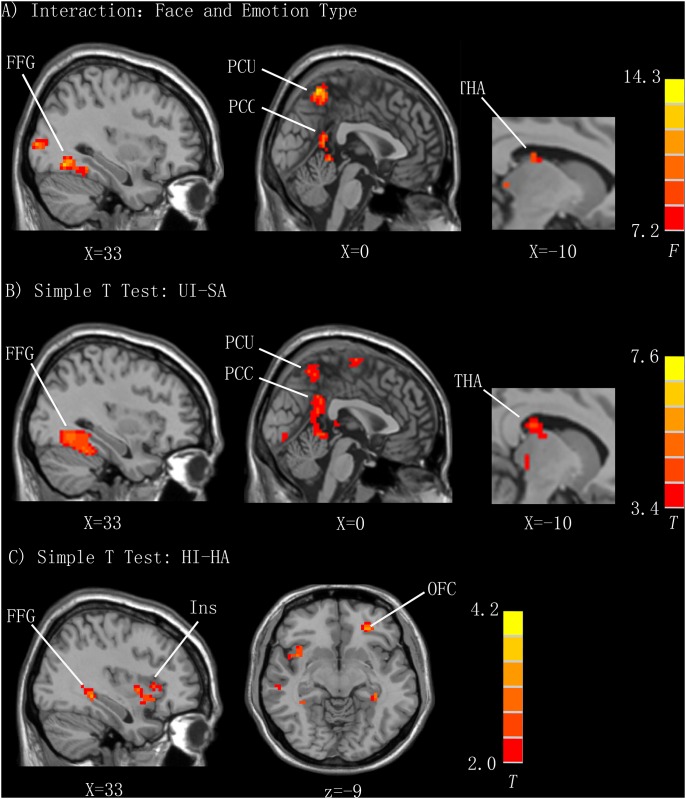
Brain regions involved in the interaction between face and emotion type and the simple t tests. A) The interaction effect between face and emotion type. The activation map is plotted with thresholds at a voxel-wise p < 0.001 (uncorrected) and an extent threshold of k = 10 for illustration. B) Simple t-test: UI-SA. Regions demonstrating enhanced activation that are not labeled are not of interest in this study. For illustration, a voxel-wise p < 0.001 (uncorrected) and an extent threshold of k = 50 is used. C) Simple t-test: HI-HA. For illustration, a voxel-wise p < 0.05 (uncorrected) and an extent threshold of k = 20 is used. FFG, fusiform gyrus; PCU, precuneus; PCC, posterior cingulate cortex; THA, thalamus; Ins, insular; OFC, orbital frontal cortex; UI, uncomfortable infant face; SA, sad adult face; HI, happy infant face; HA, happy adult face.

**Fig 4:**
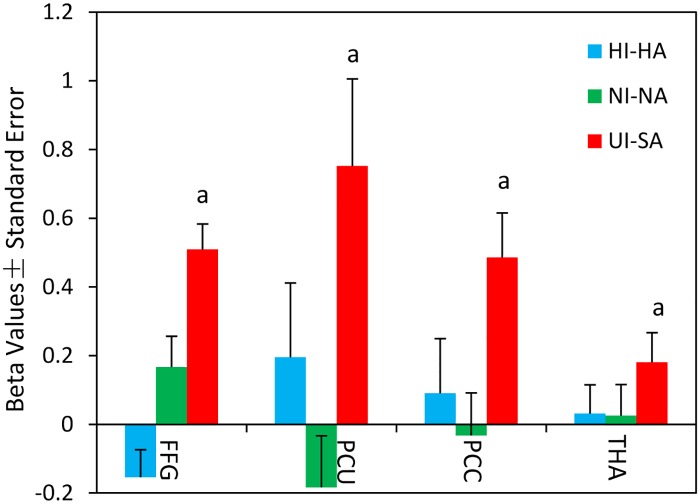
Beta values of regions of interest. Uncomfortable infant (UI) faces versus sad adult (SA) faces, but not happy or neutral infant faces versus adult faces, elicit greater activity in regions of interest (ROI). ^a^, a cluster-level FWE-corrected p < 0.05 (Voxel-wise uncorrected p < 0.001) is used. PCU, precuneus; PCC, posterior cingulate cortex; THA, thalamus; FFG, fusiform gyrus; HI, happy infant face; HA, happy adult face; NI, neutral infant face; NA, neutral adult face.

Several regions demonstrated an interaction between face and emotion types, indicating that sensitivity to face type was modulated by emotion type in these regions. These regions included the right middle occipital gyrus, with a cluster extending to the right FFG (33, -62, -10), PCU (0, -57, 60), PCC (3, -48, 6), and left THA (-10, -21, 18) (Table 2; Fig 3A). The simple-effects test demonstrated greater activation in the bilateral FFG, PCU, left THA, and PCC, with a cluster extending to the THA, evoked by uncomfortable infant faces compared to sad adult faces (Table 2, Fig 3B). Neutral infant faces activated larger brain response in the left FFG compared to neutral adult faces. Happy infant faces elicited greater activation in brain areas related to emotion and reward processing compared to happy adult faces using a more liberal threshold of p < 0.005 uncorrected (Table 2, Fig 3C). For clusters exhibiting a significant interaction, We created ROIs (6-mm radius sphere) centered on each peak coordinate and calculated individual mean contrast values of each ROI using MarsBar software [29] to facilitate interpretation. Beta values of the ROIs (right FFG, PCU, PCC, and left THA) for happy infant versus happy adult faces, neutral infant versus neutral adult faces, uncomfortable infant versus sad adult faces are plotted in Fig 4.

**Table 2:** Brain activity associated with the interaction of face and emotion type (simple-effects test).

Area	k	z Score	x	y	z
**Interaction of Face and Emotion Type**					
L Occipital Lobe/Cuneus	223	4.64	-15	-87	-12
R Middle Occipital Gyrus/FFG	272	4.56	24	-96	9
Parietal Lobe/PCU	84	4.37	0	-57	60
R PCC	38	4.14	3	-48	6
L THA	10	3.77	-10	-21	18
**Uncomfortable Infant face > Sad Adult Face**					
Occipital Lobe	1347				
R FFG		7.12	33	-62	-10
L FFG		7.64	-21	-78	-6
Sub-Lobar/THA	57	5.08	-18	-33	21
Precentral Gyrus	205	4.91	-15	-24	72
Postcentral Gyrus	46	4.78	21	-42	69
PCC-THA	217	4.58	0	-45	27
R STG	65	4.05	60	-39	15
PCU	103	4.34	0	-51	54
L STG	44	3.95	-54	-54	18
**Neutral Infant Face > Neutral Adult Face**					
L FFG	34	4.59	-21	-75	-9
**Happy Infant Face > Happy Adult Face**					
L Temporal Lobe	22	4.17	-33	-57	3
R Brain Stem	9	4.02	6	-24	-24
R Superior Frontal Gyrus	24	3.70	21	15	57
R FFG	10	3.65	33	-42	-6
L Middle Orbital Frontal Gyrus	9	3.37	-27	39	-9
R Insula cortex	13	3.22	42	3	0
R Limbic Lobe/Anterior Cingulate Cortex	15	3.00	12	27	30

Results for the interaction effect are thresholded at a peak-level FDR-corrected p < 0.05 (voxel-wise uncorrected p < 0.001) with an extent threshold of k > 10. Results for the simple effect of sad and neutral faces are thresholded at a cluster-level FWE corrected p < 0.05 (voxel-wise uncorrected p < 0.001). Results for the simple effect of happy face is thresholded at a voxel-wise p < 0.005 (uncorrected). R, right; L, left; FFG, fusiform gyrus; PCC, posterior cingulate cortex; THA, thalamus; PCU, precuneus; STG, superior temporal gyrus.

### Questionnaire Scores and Correlations with Brain Activity

**Fig 5:**
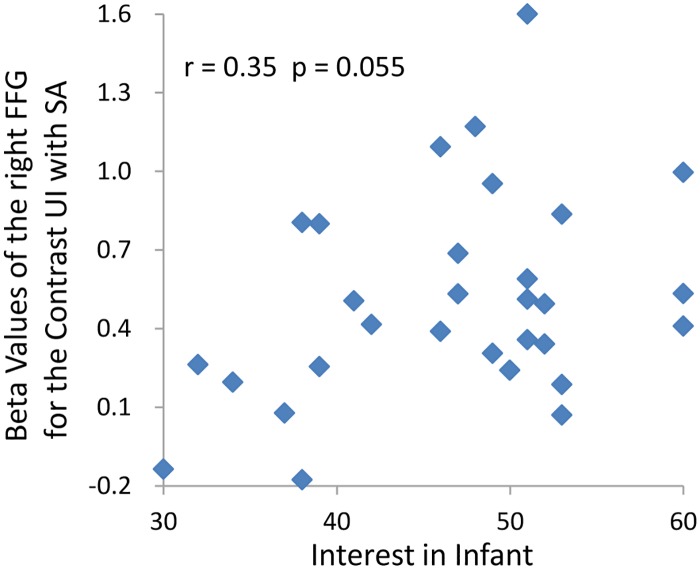
The figure only plots a marginally significant association between Interest-In-Infants and fusiform gyrus activity. For illustration, the figure only plots a marginally significant association between Interest-In-Infants scores and the beta values of FFG for uncomfortable infant faces versus sad adult faces, not for happy, neutral and uncomfortable infant faces. UI, uncomfortable infant; SA, sad adult.

**Fig 6:**
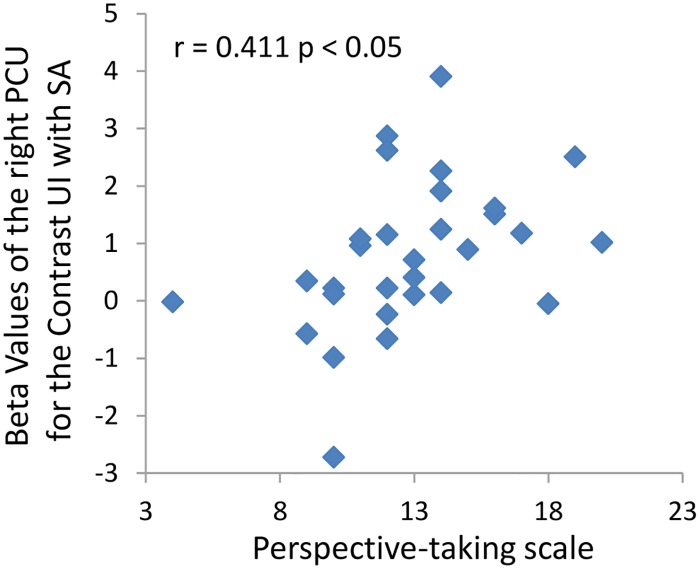
Correlation between the subscale score on Perspective Taking and precuneus activity. The figure illustrates a significant correlation between the Perspective Taking subscale scores and the beta values of PCU for uncomfortable infant faces versus sad adult faces. UI, uncomfortable infant; SA, sad adult.

We found that Interest-In-Infants was positively associated with the beta values of right FFG response to happy, neutral, and uncomfortable infant facial expression. It also associated with the contrast beta values of right FFG response to uncomfortable infant facial expression versus sad adult facial expression (Table 3, Fig 5). In addition, the Perspective Taking subscale score on the IRI-C was significantly correlated with PCU activity (r = 0.411, p < 0.05, Fig 6). Our regression model yielded a multiple correlation of R = 0.419, with an adjusted R^2^ = 0.044, F (4, 29) = 1.33, P = 0.285. While this model did not reach statistical significance; perspective taking was the only variable that approached significance, ß = 0.388, t = 1.99, p = 0.057.

**Table 3:** Pearson-correlation between the beta values of the bilateral FFG and Interest-In-Infant.

	L_FFG						R_FFG					
	HI	NI	UI	HI-HA	NI-NA	UI-SA	HI	NI	UI	HI-HA	NI-NA	UI-SA
X	0.27	0.11	0.13	0.26	-0.07	0.28	0.42*	0.42*	0.36*	0.20	0.20	**0.35**

Results for the Pearson correlations between Interest-In-Infants and the bilateral beta value of FFG. The beta vales are response to infant facial expressions and infant facial expressions compared to adult facial expressions. L, left; R, right; FFG, fusiform gyrus; X, Interest-In-Infant; HI, happy infant; NI, neutral infant; UI, uncomfortable infant; HI-HA, happy infant vs happy adult; NI-NA, neutral infant vs neutral adult; UI-SA, uncomfortable infant vs sad adult.

*significant at p < 0.05; Value in boldface is marginally significant p = 0.055.

## Discussion

The current study used fMRI to explore whether neural response is biased toward infant facial expressions compared to adult facial expressions in nulliparous women. We found that the bilateral FFG and right lingual gyrus were more activated during perception of infant faces than adult faces. We also observed regional brain response bias to uncomfortable and happy infant faces compared to sad and happy adult faces respectively. The right FFG responses to infant faces and the comparison between uncomfortable infant faces and sad adult faces were positively correlated with Interest-In-Infants. Furthermore, the PCU response bias to uncomfortable infant faces was positively correlated with Perspective Taking ability.

Greater activity in the FFG was observed when test subjects viewed infant faces compared to adult faces. Our results are consistent with previous studies, which demonstrated that the FFG plays an important role in encoding the specificity of infant faces [3, 30–32]. Importantly, We found that Interest-In-Infants was positively associated with the beta values of right FFG response to happy, neutral, and uncomfortable infant facial expression. Interest-in-Infants was also associated with the contrast beta values of right FFG response to uncomfortable infant facial expression versus sad adult facial expression. Our results may imply that the activity of the right FFG is modulated by Interest-In-Infants. A previous study reported that activity in the FFG was modulated by the OFC, which plays a role in monitoring, learning, and memory of salient reward-related stimuli in the environment [3]. Furthermore, in the dynamic interactive model of social perception, high-order social cognitive factors (e.g., stereotypes, attitudes) may be used by the OFC to implement top-down visual predictions that modulate the FFG representations of faces [17]. Taken together, our results may suggest that for females with a higher Interest-In-Infants score, infant faces are regard as motivated stimuli, attracting their visual inspection and evoking larger brain responses in the right FFG.

The contrast beta values of the right FFG were larger when comparing sad faces than for neutral and happy faces comparisons. In addition, the association between Interest-In-Infants and the contrast beta values of right FFG response to the neutral and happy faces comparisons did not reach significance. These results may be caused by no arousal difference between happy infant and adult facial expressions in our experimental stimuli, and uncomfortable infant facial expressions likely convey more biological meaning than the neutral expressions. The reasons for these results should be further investigated in future studies. The association between the left FFG activity and Interest-In-Infants was not significant, which may be caused by the differential function of the left and right FFG in face recognition [33].

We found that uncomfortable infant faces compared to sad adult faces elicited greater activation of the PCU than comparisons of happy or neutral infant versus adult faces. Consistent with our prediction, the activity in the PCU was significantly correlated with the IRI-C perspective taking factor score (cognitive empathy), but not with emotional aspects of empathy. It is known that the neuroanatomical bases of emotional and cognitive empathy are different. Specifically, the core anatomical structure associated with emotional empathy is the inferior frontal gyrus, which is implicated in emotional contagion and emotion recognition; whereas the neural bases of cognitive empathy are the ventromedial prefrontal cortex, superior temporal sulcus, temporo-parietal junction, and PCU, which are implicated in mentalizing or ToM [34, 35]. For example, the PCU is significantly more correlated with empathic accuracy in healthy controls than in schizophrenic patients demonstrating that PCU dysfunction decreases the ability of schizophrenic patients to understand others’ internal states [36]. Furthermore, the PCU is an area associated with attention [37]. Previous studies reported that infant faces elicit greater attentional engagement than adult faces, particularly when the infants displayed distress [38, 39]. An ERP study showed that smaller P300 amplitudes were elicited in mothers versus fathers, especially with infant expression of suffering, suggesting that such infant facial expressions evokes greater arousal in mothers and induces their empathic behavior [28]. Indeed, the uncomfortable infant stimuli used in this study were rated as more emotionally arousing than the sad adult stimuli. Therefore, the larger activation of the PCU while viewing uncomfortable infant faces may indicate that nulliparous women experienced both emotional and empathic responses.

The difference in PCC-THA responses observed in this study among sad, neutral, and happy faces is consistent with findings from other studies, in which greater activation in the PCC-THA was observed when mothers listened to infant cries compared to white noise [8] or when mothers viewed their own infant in a separation situation compared to a play situation [40]. MacLean [9] hypothesized that the thalamocingulate division plays a pivotal role in mammalian mother-infant attachment behavior, such as infant crying and the mother’s care-taking response. Several animal studies imply that the cingulate gyrus and its connected thalamic nuclei, which regulate attention via dopaminergic pathways, are important in mammalian maternal behavior [41]. Cingulate lesions often cause retrograde degeneration of medially located thalamic nuclei, which results in maternal behavior impairment in rats and hamsters [9, 42–44]. Therefore, activation of the PCC-THA suggests that uncomfortable infant faces elicit a care-taking response in nulliparous women.

Using a more liberal threshold, we found brain response differences in the OFC, superior frontal gyrus, ACC, and insula cortex when comparing subject responses to happy infant faces and happy adult faces. Two previous studies also reported that mothers exhibited activation of the OFC while viewing happy/positive facial expressions of their own versus unfamiliar infants [45, 46]. They concluded that the OFC may play an important role in regulating and encoding maternal attachment, and we share a similar opinion on OFC activation. Another study found that the dopaminergic reward-related regions (ventral tegmental area and striatum) were activated when mothers viewed their own infant’s happy faces compared with unknown infant’s happy faces [47]. The lack of response of the dopaminergic reward-related regions in this study may be caused by a similar level of arousal between infant and adult happy facial expressions. The other possible explanation is that the face-evoked reward circuity is modulated by sexual preference [48, 49]. Specifically, in an ERP study of heterosexual university students viewing attractive male and female adult faces, Proverbio [48] showed that both genders process opposite-sex faces differently than same-sex faces. Furthermore, Kranz [49] used fMRI to test whether subjects would respond more to their sexually preferred faces, and found that heterosexual women exhibit a significantly greater response in the thalamus and the orbitofrontal cortex when viewing male faces. Thus, adult male faces in our study might have biased the results by eliciting a greater reward-related brain response.

In the present study, unfamiliar facial expressions were used to avoid the confound of familiarity. Furthermore, the comparison of infant to adult faces was designed to extract physical features specific to infant faces. Lastly, exploring brain response to infant facial expressions among nulliparous women can help to characterize shifts in maternal brain function. However, there are some limitations to our study. Firstly, the adult face images in our experimental materials were cropped such that the forehead, which is also involved in expressing emotion, was invisible. These images will be modified to include the forehead in a future study. Secondly, some factors which may confound our results should be controlled in a future study, such as the sexual preference in the processing of face perception.

In order to determine whether the brain response bias is caused by the distinctive features of the infant face, future studies should control for the arousal level of infant and adult facial expressions. In addition, brain response to infant cues is affected by other factors, such as sex, parental status, or individual differences in parenting motivation and personality traits. For example, brain areas involved in mind wandering are modulated by sex in response to infant cries [50]. We have previously shown that childless adults with a higher secure attachment state have stronger parenting motivation [24]. Future studies should pay more attention to individual differences in the adult attachment model. The attachment model has been shown to influence women’s emotional and cognitive responses to infant expression [51–54]. Future studies should account for how the individual attachment model modulates the activation of the caregiving system in women using infant stimuli [55]. Studying this interaction between the attachment model and the caregiving system may deepen our understanding of the neural basis of women’s caregiving behavior.

In conclusion, the present study provides an examination of whether the neural response is bias toward infant facial expressions compared to adult facial expressions in nulliparous women. Our findings demonstrated that uncomfortable infant faces increase activation in regions associated with face and empathic processing compared to sad adult faces, whereas happy infant faces enhance activation in regions involved in emotion and reward processing as compared to happy adult faces using a more liberal threshold. Our findings suggest that regional brain areas associated with nulliparous women’s cognitive and emotional responses are biased toward infant facial expressions, which may be consistent with the hypothesis that the caregiving system of nulliparous women matures in the late adolescence period. Furthermore, the brain response bias to infant faces is modulated by individual differences in Interest-In-Infants and perspective taking ability.
