# Correction: Regional Brain Responses Are Biased Toward Infant Facial Expressions Compared to Adult Facial Expressions in Nulliparous Women

**DOI:** 10.1371/journal.pone.0175621

**Published:** 2017-04-06

**Authors:** The PLOS ONE Staff

The following information is missing from the Funding section: This study was supported by a grant from the National Natural Science Foundation of China (grant number: 31671149). The funder had no role in the study design, data collection and analysis, decision to publish, or preparation of the manuscript.
